# A decision tree model for hematoma expansion prediction in women after spontaneous intracerebral hemorrhage

**DOI:** 10.3389/fneur.2026.1824336

**Published:** 2026-09-07

**Authors:** Ruowen Hu, Xiaoyu Chen, Xingchen Zhou, Jinjin Liu, Yiqin Zhang

**Affiliations:** 1 Medical Quality Control Department, The First Affiliated Hospital of Wenzhou Medical University, Wenzhou, China; 2 Department of Radiology, The First Affiliated Hospital of Wenzhou Medical University, Wenzhou, China; 3 Information Department, The First Affiliated Hospital of Wenzhou Medical University, Wenzhou, China

**Keywords:** decision tree, hematoma expansion, intracerebral hemorrhage, machine learning, prediction

## Abstract

**Background:**

Women are at a higher risk of poor outcomes following spontaneous intracerebral hemorrhage (ICH) compared to men, necessitating closer clinical monitoring. Preventing hematoma expansion (HE) represents a promising therapeutic target in the management of spontaneous ICH. This study aimed to develop a clinically practical decision tree model to predict HE in women.

**Methods:**

We retrospectively reviewed women with spontaneous ICH. All patients underwent initial and follow-up non-contrast CT scans within 6 h and 72 h after symptom onset, respectively. Univariate and multivariate logistic regression analyses were used to identify independent predictors of HE. A decision tree model was developed for HE prediction.

**Results:**

A total of 417 patients were included, with 64 (15.3%) exhibiting HE on follow-up imaging. Multivariate analysis revealed that midline shift (odds ratio [OR], 1.18; 95% confidence interval [CI], 1.07–1.30; *p* = 0.001), time to initial CT scan (OR, 0.71; 95% CI, 0.57–0.88; *p* = 0.002), and presence of blend sign (OR, 2.81; 95% CI, 1.33–5.97; *p* = 0.007) were independently associated with HE. Our decision tree model achieved an AUC of 0.803 (95% CI, 0.736–0.856), a sensitivity of 81.1% and specificity of 67.1% in the training set, and 0.748 (95% CI, 0.581–0.880), 81.8 and 65.8% in the test set, respectively. It outperformed the HEP model. Although the BRAIN model had a higher AUC, our model achieved a higher sensitivity (81.8% vs. 72.7%), a key advantage for identifying patients needing timely intervention.

**Conclusion:**

We developed a simple, interpretable decision tree model to predict HE in women. This tool may support clinicians in identifying high-risk patients and guiding timely interventions.

## Introduction

Spontaneous intracerebral hemorrhage (ICH) is a life-threatening condition associated with high rates of severe disability and mortality. Following ICH, hematoma expansion (HE) occurs in approximately 39% of patients (1). HE is a well-established predictor of poor clinical outcomes and has emerged as a key target for therapeutic intervention (2). Current clinical trials, including ATACH-II (3), FAST (4), STOP-AUST (5), STOP-IT/SPOTLIGHT (6), INTERACT (7), TICH-3 (8), and FASTEST (9), have focused on preventing HE through acute blood pressure reduction or the administration of hemostatic agents.

Emerging evidence reveals uncertainty exists regarding the role of sex in risk and outcome of ICH. Previous studies highlighted persistent challenges of heterogeneity in outcome measures, potential confounders, and baseline differences in population (10, 11). Critically, a large 2025 meta-analysis (*n* = 4,812) demonstrated that despite larger hematoma volumes and a higher expansion risk in males, female sex was independently associated with worse functional outcomes after HE, − specifically, a 24% greater risks of poor functional outcomes (11, 12). These outcome disparities suggest potential sex-specific mechanisms in HE pathophysiology that cannot be fully captured by simply adjusting for sex as a covariate in mixed-cohort analyses. Therefore, a model derived from a general population may be suboptimal for women. This study aims to develop a machine learning-based prediction model tailored to HE risk stratification in women with spontaneous ICH.

## Methods

### Patient population

This study was approved by the Ethics Committee of the First Affiliated Hospital of Wenzhou Medical University (Approval number: KY2025-R313), and written informed consent was waived due to its retrospective design. We retrospectively reviewed medical records of women aged ≥ 18 years with spontaneous ICH admitted to our hospital between September 2012 and August 2018. Patients were included if they underwent an initial non-contrast CT scan within 6 h and a follow-up non-contrast CT scan within 72 h after the onset of ICH, which is compatible with routine clinical practice. Exclusion criteria included ICH secondary to intracranial aneurysm, arteriovenous malformation, traumatic brain injury, brain tumor, or hemorrhagic transformation of ischemic infarction. Additionally, patients who received antiplatelet or anticoagulant therapy or who underwent surgical intervention before the second non-contrast CT scan were excluded.

### Data collection

Upon admission, patient data including age, time from symptom onset to initial and follow-up non-contrast CT scans, medical history, and medication use were recorded. Laboratory tests were conducted to measure the following parameters: complete blood count, serum glucose, calcium, prothrombin time, activated partial thromboplastin time, prothrombin activity, fibrinogen level, and total cholesterol level. Systolic and diastolic blood pressures were measured. Neurological status was also evaluated for each patient. All imaging and clinical information was retrieved from the picture archiving and communication system.

### Radiological characterization and definition of hematoma expansion

**Figure 1:**
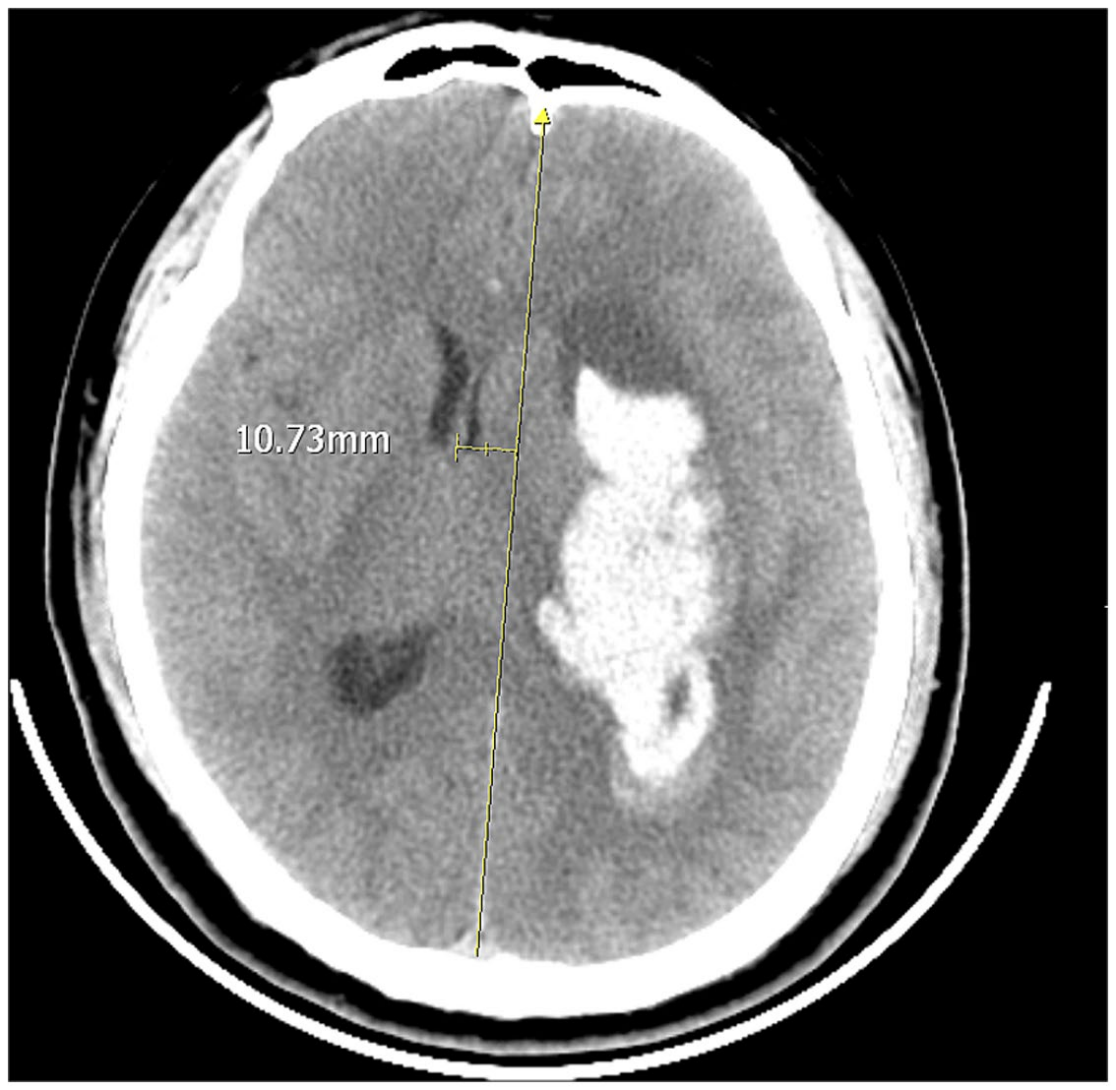
Illustration of midline shift measurement.

All patients underwent non-contrast CT scans with either a 16-channel multidetector CT scanner (BrightSpeed; GE Medical Systems, Milwaukee, WI, United States) or a 64-channel multidetector CT scanner (LightSpeed VCT 64; GE Medical Systems, Milwaukee, WI, United States), both configured with a slice thickness of 5 mm and reconstruction interval of 5 mm. The hemorrhage location, intraventricular extension, black hole sign (13), blend sign (14) and satellite sign (15) were assessed by experienced radiologists blinded to clinical information. Midline shift, maximum length, and standard deviation of the hematoma CT values were measured at the axial CT slice showing the largest hematoma area. Midline shift was assessed at three anatomical levels—the cerebral falx, septum pellucidum, and pineal gland—corresponding to the upper, middle, and lower supratentorial regions (16). An example of midline shift measurement is shown in Figure 1.

The hematoma was manually segmented on each CT slice, and the corresponding hematoma volume was calculated using the semi-automated segmentation software (3D Slicer 5.10.0). Interobserver agreement analysis for hematoma volume measurement is presented in the Supplementary File. HE was defined as an absolute increase in volume greater than 6 mL or a relative increase of more than 33% between the initial and follow-up non-contrast CT scans (1).

### Decision tree model development

Decision tree modeling was used in this study. A decision tree is a flowchart-like structure that maps outcomes based on sequential decision rules. As a nonparametric supervised machine learning method, it predicts the value of a target variable by learning simple decision rules from input data features (17). Decision trees generate interpretable classification rules, require minimal computational resources, accommodate both continuous and categorical variables, and effectively model nonlinear relationships.

The entire dataset was randomly partitioned into training and test sets in an 8:2 ratio. Recursive feature elimination (RFE) technique was applied to the training set for feature selection, yielding six key variables: midline shift, time to initial non-contrast CT scan, initial hematoma volume, prothrombin activity, blend sign, and Glasgow Coma Scale (GCS) score. RFE technique is a purely data-driven feature selection procedure. The multivariable logistic regression was performed to identify independent predictors and to quantify the strength of their associations with HE (using odds ratios) – this is an explanatory, hypothesis-testing exercise. It is RFE, not the regression results, guided the selection of candidate variables for the decision tree. Hyperparameter tuning was performed using randomized search strategy with five-fold cross-validation. The final decision tree model was configured with the following hyperparameters: class weight, “balanced”; impurity criterion, “Gini”; maximum tree depth, 4; maximum feature, 6; minimum samples per leaf, 10; minimum samples per split, 15; and splitter, “best.” Model development was implemented using Python (version 3.8; Python Software Foundation), along with Scikit-learn (version 0.24.1; Scikit-learn Developers).

Model performance was evaluated using sensitivity and specificity. Sensitivity was defined as the proportion of hematoma expanders that were correctly identified by the model. Specificity was defined as the proportion of non-expanders that were accurately classified.

### Statistical analysis

Statistical analyses were conducted using IBM SPSS (version 22.0; IBM Corp., Armonk, New York, United States). Continuous variables are expressed as mean ± standard deviation (SD), and categorical variables as frequency (percentages). Intergroup comparisons for continuous variables were assessed using the Student’s *t*-test or Mann–Whitney *U* test, and for categorical variables using the *χ*^2^ or Fisher’s exact tests, as appropriate. Variables with a *p*-value less than 0.1 were included in the multivariable logistic regression analysis. Multivariate logistic regression analysis with a forward method was performed to identify factors independently associated with HE. Variables with more than 5% missing data were excluded from the multivariate logistic regression analysis. DeLong test was performed to compare the AUCs between the decision tree model and each of the other two models [HEP (18) and BRAIN (19)]. Statistical significance was set at *p* < 0.05.

## Results

A total of 417 patients with ICH were enrolled, of whom 64 (15.3%) experienced HE. The mean ages of expanders and non-expanders were 62.3 ± 11.6 and 62.7 ± 12.7 years, respectively (*p* = 0.907). Among patients with HE, hemorrhage was located in the deep gray matter in 52 cases (76.5%), lobar region in 7 (10.3%), the brain stem in 5 (7.4%), cerebellum in 2 (2.9%), and in multiple locations in 2 (2.9%).

Table 1 presents the results of univariate analysis comparing expanders and non-expanders. Compared with non-expanders, expanders had a significantly shorter time from symptom onset to the initial non-contrast CT scan (2.6 ± 1.3 vs. 3.1 ± 1.5 h, *p* = 0.002), lower GCS score (11.1 ± 3.4 vs. 12.2 ± 3.2, *p* = 0.013), and larger hematoma volume on the initial non-contrast CT scan (23.90 ± 17.38 vs. 17.7 ± 14.0 mL, *p* = 0.008). Two laboratory parameters—prothrombin activity and aspartate transaminase— showed statistically significant differences between the two groups. The blend sign, an imaging marker, was more frequently observed in expanders than in non-expanders. The midline shift was greater in expanders compared to non-expanders. The deep gray matter was the most common hemorrhage location in both groups; however, no statistically significant difference was found between expanders and non-expanders. Multivariate logistic regression analysis (Table 2) identified midline shift, time to initial non-contrast CT scan, and blend sign as independent predictors of HE.

**Table 1:** Comparisons of variables between expanders and non-expanders baseline characteristics.

Clinical variables	Non-expanders (*n* = 353)	Expanders (*n* = 64)	*p*-value
Age (years)	62.7 ± 12.7	62.3 ± 11.6	0.907
Glasgow Coma Scale score^*^	12.2 ± 3.2	11.1 ± 3.4	0.013
Reexamine time (h)	23.0 ± 16.3	19.6 ± 14.8	0.144
Baseline hematoma volume (mL)	17.7 ± 14.0	23.90 ± 17.38	0.008
Systolic pressure^†^	161.3 ± 25.0	151.6 ± 25.5	0.009
Diastolic pressure^†^	88.2 ± 14.7	85.1 ± 17.6	0.157
Pressure difference^†^	73.1 ± 20.1	66.6 ± 16.4	0.023
History of hemorrhage	11 (3.1%)	4 (6.3%)	0.263
History of infarction	17 (4.8%)	1 (1.6%)	0.331
History of diabetes mellitus	48 (13.6%)	6 (9.4%)	0.355
History of hypertension	272 (77.1%)	46 (71.9%)	0.370
History of hyperlipidemia	42 (11.9%)	4 (6.3%)	0.185
Coronary disease	4 (1.1%)	0 (0.0%)	NA
Laboratory tests			
Ca^‡^	2.24 ± 0.11	2.26 ± 0.12	0.271
Glucose (mmol/L)	8.13 ± 2.91	8.38 ± 3.72	0.552
Neutrophil %	0.75 ± 0.13	0.74 ± 0.13	0.363
Eosinophil	0.06 ± 0.08	0.08 ± 0.14	0.461
Neutrophil (X10^9^/L)	7.59 ± 3.69	7.12 ± 3.77	0.260
Mononuclear (X10^9^/L)	0.50 ± 0.30	0.44 ± 0.17	0.122
lymphocyte	1.49 ± 0.91	1.61 ± 0.92	0.478
Basophilic granulocyte (X10^9^/L)	0.02 ± 0.04	0.02 ± 0.04	0.341
Red blood cell (X10^12^/L)	4.30 ± 0.49	4.32 ± 0.52	0.983
White blood cell (X10^9^/L)	9.68 ± 3.55	9.26 ± 3.73	0.315
Platelet (X10^9^/L)	216 ± 78	205 ± 63	0.249
Hemoglobin (g/L)^‡^	130 ± 15	130 ± 19	0.727
Prothrombin time (second)^‡^	13.2 ± 1.2	13.5 ± 2.4	0.192
Prothrombin activity (%)^‡^	101.8 ± 14.0	99.6 ± 15.7	0.031
International normalized ratio^‡^	1.00 ± 0.12	1.04 ± 0.27	0.073
Fibrinogen (g/L)^‡^	3.52 ± 0.88	3.33 ± 0.86	0.410
APPT (second)^‡^	34.2 ± 5.5	34.7 ± 5.0	0.137
APPT ratio^‡^	0.95 ± 0.14	0.95 ± 0.17	0.171
Thrombin time (second)^‡^	16.6 ± 1.4	16.6 ± 1.1	0.979
Thrombin time ratio^‡^	0.98 ± 0.11	0.98 ± 0.06	0.584
Albumin (g/L)	39.91 ± 6.96	39.70 ± 6.56	0.243
Alanine aminotransferase (U/L)^§^	27 ± 27	25.90 ± 15.02	0.538
Aspartate transaminase (U/L)^||^	28 ± 16	30.18 ± 14.33	0.042
Total cholesterol (mmol/L)^**^	5.48 ± 1.29	5.33 ± 1.03	0.444
Triglycerides (mmol/L)^**^	1.74 ± 2.54	1.72 ± 1.24	0.753
HDL-C (mmol/L)^**^	1.33 ± 0.77	1.30 ± 0.31	0.658
LDL-C (mmol/L)^**^	3.31 ± 1.07	3.12 ± 0.82	0.263
Radiological characteristics			
Time to initial CT (hour)	3.1 ± 1.5	2.6 ± 1.3	0.002
Location of hemorrhage			0.546
Deep gray matter	298 (84.4%)	52 (76.5%)	
Lobar region	28 (7.9%)	7 (10.3%)	
Brain stem	17 (4.8%)	5 (7.4%)	
Cerebellum	7 (2.0%)	2 (2.9%)	
Multiple locations	3 (0.8%)	2 (2.9%)	
Intraventricular extension	131(37.1%)	25 (39.1%)	0.767
Black hole sign	50 (14.2%)	14 (21.9%)	0.115
Blend sign	28 (7.9%)	13 (20.3%)	0.002
Satellite sign	180 (51.0%)	36 (56.3%)	0.439
Subarachnoid hemorrhage	27 (7.6%)	4 (6.3%)	0.376
SD of hematoma CT values (Hu)	8.1 ± 2.0	8.1 ± 2.4	0.724
Midline shift (mm)	3.52 ± 2.66	4.79 ± 2.95	0.001
Maximum length (mm)	38.99 ± 13.62	43.81 ± 14.38	0.010

^*^9/417 (2.2%) missing values.

^†^52/417 (12.5%) missing values.

^‡^3/417 (<1.0%) missing values.

^§^32/417 (7.7%) missing values.

^||^36/417 (8.6%) missing values.

^**^61/417 (14.6%) missing values.

APPT, activated partial thromboplastin time; LDL-C, low density lipoprotein cholesterol; HDL-C, high density lipoprotein cholesterol; SD, standard deviation.

**Table 2:** Results of multivariate logistic regression analysis.

Variable	*β* coefficient	OR	95% CI	*P*-value
Midline shift	0.16 ± 0.05	1.18	1.07–1.30	0.001
Time to initial CT scan	−0.334 ± 0.11	0.71	0.57–0.88	0.002
Blend sign	1.03 ± 0.38	2.81	1.33–5.97	0.007

OR, odds ratio; CI, confidence interval.

**Figure 2:**
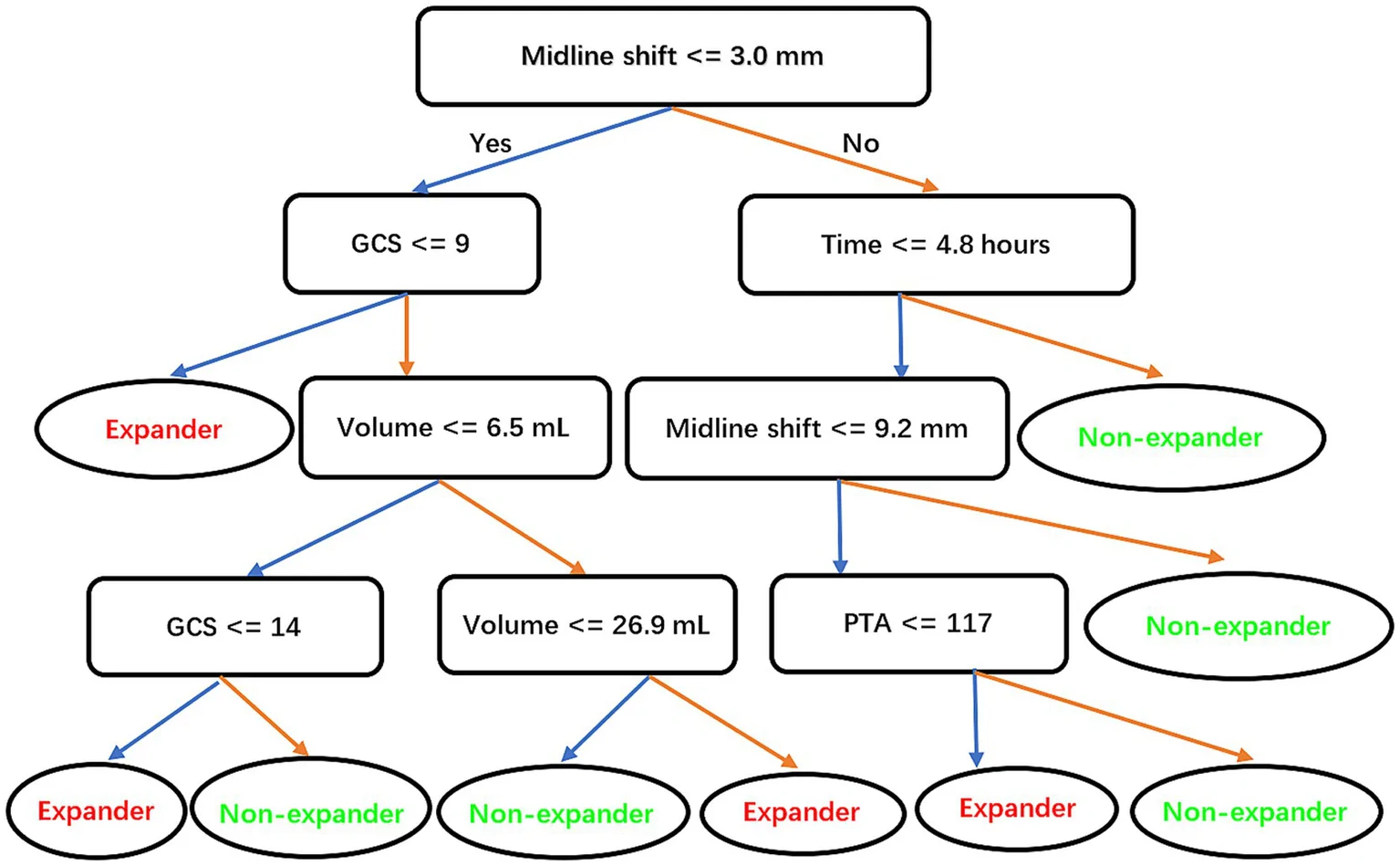
Decision tree model for hematoma expansion prediction. In the decision tree, the rectangle node represents condition, and the elliptical node means the predicted outcome, i.e., expander or non-expander. One can start from the root node, “Midline shift <= 3.0 mm,” and compare the value a patient’s midline shift with the condition in the root node. If it is true that the patient’s midline shift is less or equal to 3.0 mm, then moved to the next node, “GCS < 9”; otherwise, jump to the node of “Time <= 4.8 h.” Continue comparing patient’s attribute value with other internal nodes of the tree until an elliptical node is reached, at which point the predicted outcome is provided. Volume in the decision tree indicates initial hematoma volume; GCS = Glasgow Coma Scale; PTA = Prothrombin activity. Observed ranges for continuous variables include midline shift (0–12.85 mm), time to initial CT (0.5–6.0 h), initial volume (0.3–91.0 mL), prothrombin activity (28 – 150%), and GCS (3 – 15). These ranges reflect the distribution of our dataset; the tree’s decision rules are most reliable when applied within or near these ranges. For patients with values falling substantially outside these boundaries, predictions should be interpreted with caution and integrated with overall clinical judgment.

**Figure 3:**
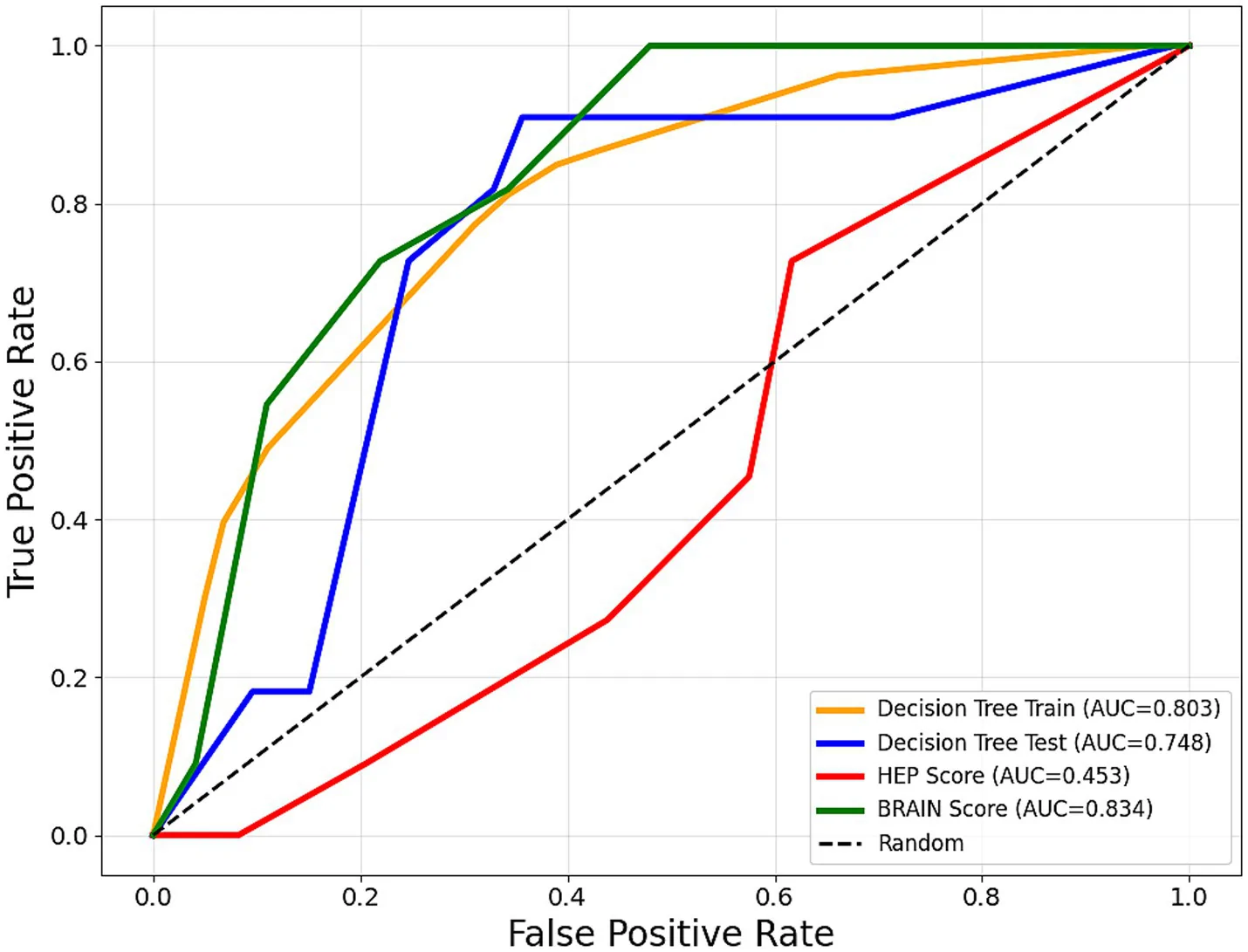
Receiver operating characteristic curves of decision tree, HEP, and BRAIN models.

Figure 2 illustrates the decision tree model, which incorporated five attributes for HE prediction: midline shift, GCS score, time to initial non-contrast CT scan, initial hematoma volume, and prothrombin activity. Detailed instructions for applying this model are provided in the legend of Figure 2. Table 3 demonstrates the comparison of prediction results. Figure 3 shows the ROC curves. Our decision tree model achieved an AUC of 0.803 (95% CI, 0.736–0.856), a sensitivity of 81.1%, and a specificity of 67.1% in the training dataset. In the test dataset, the AUC was 0.748 (95% CI, 0.581–0.880), with a sensitivity of 81.8% and a specificity of 65.8%. For comparison, the HEP model from the literature yielded an AUC of 0.453 (95% CI, 0.305–0.610), a sensitivity of 45.5%, and a specificity of 42.5%; the BRAIN model showed an AUC of 0.834 (95% CI, 0.727–0.923), a sensitivity of 72.7%, and a specificity of 78.0%. DeLong tests indicated that the decision tree model outperformed the HEP model (*p* < 0.001). The difference between decision tree and the BRAIN model was also statistically significant (*p* = 0.002), with the Brain model showing a higher AUC (Table 4).

**Table 3:** Prediction results of decision tree Model for (a) training and (b) test datasets.

Actual status	Predicted status		
	Non-expander	Expander	% Correct
(a)			
Non-expander	188	92	67.1%
Expander	10	43	81.1%
(b)			
Non-expander	48	25	65.8%
Expander	2	9	81.8%

**Table 4:** Performance of decision tree model in the training and test datasets.

Models	AUC (95% CI)	Sensitivity	Specificity
DT* in training	0.803 (0.736–0.856)	0.811	0.671
DT in test	0.748 (0.581–0.880)	0.818	0.658
HEP	0.453 (0.305–0.610)	0.455	0.425
BRAIN	0.834 (0.727–0.923)	0.727	0.780

^*^DT, decision tree.

## Discussion

In this study, we investigated the risk factors for HE in women following spontaneous ICH. Furthermore, we developed a clinically valuable and user-friendly decision tree model with high predictive sensitivity, which may facilitate clinical decision-making.

Although in the acute phase of ICH, male patients typically exhibit more severe clinical features, including deeper hemorrhage, larger baseline hematoma volume, and higher risk of hematoma expansion, a meta-analysis (12) shows that the long-term functional prognosis of females is actually worse. This seemingly contradictory phenomenon may be explained by the following factors: First, female experienced ICH are generally older than male patients (12), and advanced age itself is a powerful negative predictor of neurological function recovery. Second, sex-based disparities in treatment and care intensity may exist: observational data suggest that males tend to receive more aggressive interventions, such as higher rates of mechanical ventilation, renal replacement therapy, and prolonged ICU stays (20). Additionally, comorbidities more prevalent in women, such as osteoporosis and frailty (21), may constrain rehabilitation potential. In conclusion, the inferior prognosis observed in females appears to reflect not only the initial hemorrhage severity but also the influence of age, sociobehavioral factors, and unmeasured comorbidities. Of note, although prior studies have associated sex with post-ICH outcomes, a recent study (22) found no significant association between gonadal hormone levels and clinical outcomes. Further investigation is warranted.

Our decision tree model incorporated five features for HE prediction: midline shift, time to initial non-contrast CT scan, GCS score, initial hematoma volume, and prothrombin activity. These features have been confirmed to be significant predictors of HE. The presence of midline shift reflects elevated intracranial pressure and is associated with worse prognosis (23). The incidence of hematoma growth decreases progressively with longer intervals between symptom onset and the initial non-contrast CT scan, since delayed presentation often coincides with stabilized hematoma formation by the time of imaging (24). Impaired consciousness at admission, reflected by lower GCS scores, is strongly linked to higher risks of HE (24, 25). Additionally, admission levels of coagulation markers, such as prothrombin activity, serve as valuable predictors of HE (26), and maintaining these parameters within normal ranges has emerged as a potential therapeutic strategy in ICH management (27). Numerous studies (24, 28) have consistently showed that initial hematoma volume is significantly greater in patients who experience expansion compared to those who do not. It was noted that the ‘blend sign’, identified as a significant independent predictor in the multivariable logistic regression analysis, did not appear as a splitting node in the final pruned decision tree. This omission is attributable to the deliberate imposition of the ‘max_depth = 4’ hyperparameter constraint—introduced to prioritize model interpretability, enhance generalizability, and mitigate overfitting—thereby limiting the tree’s capacity to incorporate all statistically significant variables within its hierarchical structure. Although the regression and the decision tree share some variables, they are not sequentially dependent; they are complementary analytical tools: one provides epidemiological inference, the other provides a practical risk-stratification rule.

Clinical scoring tools have been developed for HE prediction. Representative tools include the five-point BAT score (29), the 3-predictor model (30), the 9-point score model (31), the HEP score model (18), the HEAVN scale model (32), the NAG scale model (33), and the 24-point BRAIN score derived from the INTERACT trials involving multiple hospitals across 21 countries (19). The BRAIN score demonstrated good discrimination and calibration (19). With advances in modern artificial intelligence, machine learning algorithms have increasingly been applied to HE prediction. A systematic review indicates that radiomics models outperform clinical models in discriminating HE, with C-indices of 0.77 and 0.73 in the training and validation cohorts, respectively, compared to 0.69 and 0.70 for clinical models (34). A recent meta-analysis (35) reported pooled sensitivity of 0.81 and 0.84, specificity of 0.79 and 0.91, and area under the curve of 0.87 and 0.89 for exclusive deep learning-based and combined deep learning-based models, respectively. In our comparative analysis, the decision tree model achieved a significantly higher AUC than the HEP score (0.748 vs. 0.453), but a lower AUC compared to the BRAIN score (0.748 vs. 0.834). However, our model achieved a sensitivity of 81.8% on the test dataset, which means the model can correctly identify most patients who truly need intervention for hematoma expansion. Notably, the model is simple and easily interpretable, enhancing its potential for integration into clinical decision-making processes.

This study has several limitations. First, this is a single-center retrospective analysis. Second, our model has not been validated using external datasets from other institutions, potentially restricting its generalizability to broader patient populations. Third, prehospital deaths and those who missed the second non-contrast CT scan were excluded from this study, which could have introduced inclusion bias and affected the representativeness of the study cohort. Finally, the decision tree, by its nature, impose rigid-cut-offs that may produce counterintuitive classifications at the extremes. Our model is meant as an aid, not a replacement for clinical assessment, and that future work with larger datasets could help define more robust boundaries. Nevertheless, the proposed decision tree model demonstrates good discriminative performance for HE prediction, can be implemented with relative ease in clinical settings, and may serve as a practical tool to support treatment decision-making.

In summary, we developed a simple and clinically applicable decision tree model for HE prediction in women. Women are at higher risk of poor outcomes following spontaneous ICH, warranting close clinical attention. Our model may assist in identifying candidates who are most likely to benefit from interventions targeting HE reduction and may provide valuable diagnostic insights for clinicians, patients, and their families.

## Data Availability

The raw data supporting the conclusions of this article will be made available by the authors, without undue reservation.

## References

[1] Mutimer CA Wu TY Zhao H Churilov L Campbell BCV Cheung A . Ultra-early hematoma expansion is associated with ongoing hematoma growth and poor functional outcome. Stroke. (2025) 56:838–47. doi: 10.1161/STROKEAHA.124.050131, 39882606

[2] Morotti A Boulouis G Nawabi J Li Q Charidimou A Pasi M . Association between hematoma expansion severity and outcome and its interaction with baseline intracerebral hemorrhage volume. Neurology. (2023) 101:e1606–13. doi: 10.1212/WNL.0000000000207728, 37604661 PMC10585678

[3] Qureshi AI Palesch YY. Antihypertensive treatment of acute cerebral hemorrhage (ATACH) II: design, methods, and rationale. Neurocrit Care. (2011) 15:559–76. doi: 10.1007/s12028-011-9538-3, 21626077 PMC3340125

[4] Mayer SA Brun NC Begtrup K Broderick J Davis S Diringer MN . Efficacy and safety of recombinant activated factor VII for acute intracerebral hemorrhage. N Engl J Med. (2008) 358:2127–37. doi: 10.1056/NEJMoa0707534, 18480205

[5] Meretoja A Churilov L Campbell BC Aviv RI Yassi N Barras C . The spot sign and tranexamic acid on preventing ICH growth--AUStralasia trial (STOP-AUST): protocol of a phase II randomized, placebo-controlled, double-blind, multicenter trial. Int J Stroke. (2014) 9:519–24. doi: 10.1111/ijs.12132 23981692

[6] Gladstone DJ Aviv RI Demchuk AM Hill MD Thorpe KE Khoury JC . Effect of recombinant activated coagulation factor VII on hemorrhage expansion among patients with spot sign-positive acute intracerebral hemorrhage: the SPOTLIGHT and STOP-IT randomized clinical trials. JAMA Neurol. (2019) 76:1493–501. doi: 10.1001/jamaneurol.2019.2636, 31424491 PMC6704754

[7] Anderson CS Huang Y Wang JG Arima H Neal B Peng B . Intensive blood pressure reduction in acute cerebral haemorrhage trial (INTERACT): a randomised pilot trial. Lancet Neurol. (2008) 7:391–9. doi: 10.1016/S1474-4422(08)70069-3, 18396107

[8] Law ZK Menon CS Woodhouse LJ Appleton JP Al-Shahi Salman R Robinson T . Outcome 1 year after ICH: data from the tranexamic acid for IntraCerebral Haemorrhage 2 (TICH-2) trial. Eur Stroke J. (2025) 10:206–15. doi: 10.1177/23969873241265939, 39076020 PMC11569519

[9] Naidech AM Grotta J Elm J Janis S Dowlatshahi D Toyoda K . Recombinant factor VIIa for hemorrhagic stroke treatment at earliest possible time (FASTEST): protocol for a phase III, double-blind, randomized, placebo-controlled trial. Int J Stroke. (2022) 17:806–9. doi: 10.1177/17474930211042700, 34427473 PMC9933458

[10] Gokhale S Caplan LR James ML. Sex differences in incidence, pathophysiology, and outcome of primary intracerebral hemorrhage. Stroke. (2015) 46:886–92. doi: 10.1161/strokeaha.114.007682, 25657181

[11] Marini S Morotti A Ayres AM Crawford K Kourkoulis CE Lena UK . Sex differences in intracerebral hemorrhage expansion and mortality. J Neurol Sci. (2017) 379:112–6. doi: 10.1016/j.jns.2017.05.057, 28716219 PMC5538146

[12] Rivier CA Renedo D Marini S Magid-Bernstein JR de Havenon A Rosand J . Sex modifies the severity and outcome of spontaneous intracerebral hemorrhage. Ann Neurol. (2025) 97:232–41. doi: 10.1002/ana.27123 39499118

[13] He GN Guo HZ Han X Wang EF Zhang YQ. Comparison of CT black hole sign and other CT features in predicting hematoma expansion in patients with ICH. J Neurol. (2018) 265:1883–90. doi: 10.1007/s00415-018-8932-6, 29948249

[14] Li Q Zhang G Huang YJ Dong MX Lv FJ Wei X . Blend sign on computed tomography: novel and reliable predictor for early hematoma growth in patients with intracerebral hemorrhage. Stroke. (2015) 46:2119–23. doi: 10.1161/STROKEAHA.115.009185 26089330

[15] Shimoda Y Ohtomo S Arai H Okada K Tominaga T. Satellite sign: a poor outcome predictor in intracerebral hemorrhage. Cerebrovasc Dis. (2017) 44:105–12. doi: 10.1159/000477179, 28605739

[16] Yang WS Li Q Li R Liu QJ Wang XC Zhao LB . Defining the optimal midline shift threshold to predict poor outcome in patients with Supratentorial spontaneous intracerebral hemorrhage. Neurocrit Care. (2018) 28:314–21. doi: 10.1007/s12028-017-0483-7, 29139015

[17] Charbuty B Abdulazeez A. Classification based on decision tree algorithm for machine learning. J Appl Sci Technol Trends. (2021) 2:20–8. doi: 10.38094/jastt20165

[18] Yao X Xu Y Siwila-Sackman E Wu B Selim M. The HEP score: a nomogram-derived hematoma expansion prediction scale. Neurocrit Care. (2015) 23:179–87. doi: 10.1007/s12028-015-0147-4, 25963292

[19] Wang X Arima H Al-Shahi Salman R Woodward M Heeley E Stapf C . Clinical prediction algorithm (BRAIN) to determine risk of hematoma growth in acute intracerebral hemorrhage. Stroke. (2015) 46:376–81. doi: 10.1161/STROKEAHA.114.006910, 25503550

[20] Modra LJ Higgins AM Abeygunawardana VS Vithanage RN Bailey MJ Bellomo R. Sex differences in treatment of adult intensive care patients: a systematic review and meta-analysis. Crit Care Med. (2022) 50:913–23. doi: 10.1097/ccm.0000000000005469, 35148525

[21] Liang H Chen S Shi M Xu J Zhao C Yang B . Global epidemiology and burden of osteoporosis among postmenopausal women: insights from the global burden of disease study 2021. npj Aging. (2025) 11:78. doi: 10.1038/s41514-025-00269-2, 40890217 PMC12402057

[22] Ng Y Qi W Covington A Boone B Kuhn C Nixon AB . Pilot exploratory analysis of serum gonadal hormones, inflammatory proteins, and intracerebral hemorrhage outcomes. Int J Mol Sci. (2025) 26:8334. doi: 10.3390/ijms26178334, 40943256 PMC12427954

[23] Liao C-C Chen Y-F Xiao F. Brain midline shift measurement and its automation: a review of techniques and algorithms. Int J Biomed Imaging. (2018) 2018:1–13. doi: 10.1155/2018/4303161, 29849536 PMC5925103

[24] Fujii Y Takeuchi S Sasaki O Minakawa T Tanaka R. Multivariate analysis of predictors of hematoma enlargement in spontaneous intracerebral hemorrhage. Stroke. (1998) 29:1160–6. doi: 10.1161/01.str.29.6.1160 9626289

[25] Li Q Zhang G Xiong X Wang XC Yang WS Li KW . Black hole sign: novel imaging marker that predicts hematoma growth in patients with intracerebral hemorrhage. Stroke. (2016) 47:1777–81. doi: 10.1161/STROKEAHA.116.013186, 27174523

[26] Takahashi H Urano T Nagai N Takada Y Takada A. Progressive expansion of hypertensive intracerebral hemorrhage by coagulopathy. Am J Hematol. (1998) 59:110–4. doi: 10.1002/(sici)1096-8652(199810)59:2<>3.0.co;2-0, 9766794

[27] McBride D Tang J Zhang JH. Maintaining plasma fibrinogen levels and fibrinogen replacement therapies for treatment of intracranial hemorrhage. Curr Drug Targets. (2017) 18:1349–57. doi: 10.2174/1389450117666151209123857, 26648060

[28] Fu J Hu S Yang M Li Z Song X Wang Z . A novel 10-point score system to predict early hematoma growth in patients with spontaneous intracerebral hemorrhage. Front Neurol. (2019) 10:1417. doi: 10.3389/fneur.2019.01417, 32116989 PMC7018853

[29] Morotti A Dowlatshahi D Boulouis G Al-Ajlan F Demchuk AM Aviv RI . Predicting intracerebral hemorrhage expansion with noncontrast computed tomography: the BAT score. Stroke. (2018) 49:1163–9. doi: 10.1161/STROKEAHA.117.020138, 29669875 PMC6034631

[30] Takeda R Ogura T Ooigawa H Ooigawa H Fushihara G Fushihara G . A practical prediction model for early hematoma expansion in spontaneous deep ganglionic intracerebral hemorrhage. Clin Neurol Neurosurg. (2013) 115:1028–31. doi: 10.1016/j.clineuro.2012.10.016 23245855

[31] Brouwers HB Chang Y Falcone GJ Cai X Ayres AM Battey TW . Predicting hematoma expansion after primary intracerebral hemorrhage. JAMA Neurol. (2014) 71:158–64. doi: 10.1001/jamaneurol.2013.5433, 24366060 PMC4131760

[32] Miyahara M Noda R Yamaguchi S Tamai Y Inoue M Okamoto K . New prediction score for hematoma expansion and neurological deterioration after spontaneous intracerebral hemorrhage: a hospital-based retrospective cohort study. J Stroke Cerebrovasc Dis. (2018) 27:2543–50. doi: 10.1016/j.jstrokecerebrovasdis.2018.05.018, 29880210

[33] Sakuta K Sato T Komatsu T Sakai K Terasawa Y Mitsumura H . The NAG scale: Noble predictive scale for hematoma expansion in intracerebral hemorrhage. J Stroke Cerebrovasc Dis. (2018) 27:2606–12. doi: 10.1016/j.jstrokecerebrovasdis.2018.05.020, 29958849

[34] Liu Y Zhao F Niu E Chen L. Machine learning for predicting hematoma expansion in spontaneous intracerebral hemorrhage: a systematic review and meta-analysis. Neuroradiology. (2024) 66:1603–16. doi: 10.1007/s00234-024-03399-8, 38862772

[35] Ahmadzadeh AM Ashoobi MA Broomand Lomer N Elyassirad D Gheiji B Vatanparast M . Application of deep learning for predicting hematoma expansion in intracerebral hemorrhage using computed tomography scans: a systematic review and meta-analysis of diagnostic accuracy. Radiol Med. (2025) 130:1973–85. doi: 10.1007/s11547-025-02089-6, 40932678

