# Supplementary material for: A decision tree model for hematoma expansion prediction in women after spontaneous intracerebral hemorrhage
Source: Front Neurol. 2026 Sep 7;17:1824336. doi: 10.3389/fneur.2026.1824336 (PMC13590341; doi:10.3389/fneur.2026.1824336)
Supplement: Supplementary file 1 [file Table_1.docx]

**Interobserver Agreement Analysis for Hematoma Volume Measurement**

**Purpose:** To assess the interobserver reliability and reproducibility of hematoma volume measurements.

**Methods:**

We randomly selected 20 patients for hematoma volume measurement on non-contrast CT scans. Hematoma volumes were measured independently by the two experienced radiologists, who were blinded to each other's measurements and all clinical data. The software's semi- automated segmentation algorithm was applied, with manual correction by the radiologist to ensure accurate delineation of the hematoma margins on each axial slice. The final volume was calculated automatically by the software.

The interobserver agreement for the absolute hematoma volumes was assessed using a two-way random-effects model for the Intraclass Correlation Coefficient (ICC), estimating absolute agreement for single measures (ICC(2,1)). 95% confidence intervals (CI) were calculated.

**Results:**

The volume measurements between the two radiologists are presented in the supplementary Table 1. The interobserver reliability for hematoma volume measurements was excellent. The ICC(2,1) for all measured volumes was 0.960 (95% CI: [0.903 – 0.984]).

Supplementary Table 1. Detailed volume (mL) measurements between two radiologists

| Sample Number | Volume measured by radiologist 1 | Volume measured by radiologist 2 |
| --- | --- | --- |
| 1 | 23.67 | 24.35 |
| 2 | 79.43 | 91.87 |
| 3 | 25.46 | 26.67 |
| 4 | 75.68 | 78.33 |
| 5 | 26.96 | 27.8 |
| 6 | 25.81 | 28.47 |
| 7 | 20.14 | 21.61 |
| 8 | 49.98 | 26.19 |
| 9 | 62.66 | 59.88 |
| 10 | 12.63 | 13.39 |
| 11 | 41.42 | 39.69 |
| 12 | 45.05 | 44.95 |
| 13 | 92.72 | 100.59 |
| 14 | 40.95 | 42.87 |
| 15 | 82.49 | 87.75 |
| 16 | 18.65 | 24.16 |
| 17 | 15.67 | 19.32 |
| 18 | 27.25 | 30.62 |
| 19 | 53.60 | 64.85 |
| 20 | 12.5 | 20.95 |

**Conclusion:**

The independent hematoma volume measurements performed by two experienced radiologists showed good interobserver agreement. This high level of reproducibility confirms that our method for hematoma volume segmentation is accurate and reliable.
